# Research on Developing an Outdoor Location System Based on the Ultra-Wideband Technology

**DOI:** 10.3390/s20216171

**Published:** 2020-10-29

**Authors:** Łukasz Rykała, Andrzej Typiak, Rafał Typiak

**Affiliations:** Faculty of Mechanical Engineering, Military University of Technology, 2 gen. S. Kaliskiego str., 00-908 Warsaw, Poland; andrzej.typiak@wat.edu.pl (A.T.); rafal.typiak@wat.edu.pl (R.T.)

**Keywords:** ultra-wideband, UWB, localization, trilateration, nonlinear programming, NLP, follow me, unmanned ground vehicles, UGV, outdoor location system

## Abstract

Ultra-wideband (UWB) technology is one of the most promising wireless communication technologies. Examples of UWB applications include, among others, radiocommunication devices and location systems, due to their operating range, ability to work in outdoor environments, and resistance to multipath effects. This article focuses on the use of UWB technology in constructing a guide localization system for an unmanned ground vehicle (UGV), which is one of the stages of implementing a “follow me” system. This article describes the complete process of UWB signal processing from its acquisition, methods of filtering, and obtained results, to determining the location of the guide. This article examines the possibility of using modified versions of localization algorithms for determining the guide’s location, including trilateration, methods of nonlinear programming, and a geometric algorithm proposed by us. The innovation of this study consists in the implementation of an algorithm that changes the selection of equations (mathematical model) for determining location based on the number of available measurements from UWB sensors.

## 1. Introduction

Unmanned ground vehicles (UGVs) are robotic systems that operate on land without an onboard human operator. They can be used in both civil and military applications. Increasingly, more UGVs are being used to perform tasks considered dangerous for people, especially in outdoor environments, such as firefighting or Improvised Explosive Device (IED) neutralization. In addition, they are being used to patrol borders, evacuate wounded soldiers on the battlefield, carry equipment, forward reconnaissance, and act as mobile weapon platforms. The aforementioned applications have emerged from the current trends in the design of UGVs, which focus on the protection of human life and assume that these tasks will ultimately be carried out solely by machines [1,2,3,4].

Depending on their application, UGVs can be controlled remotely by an operator either from the immediate platform’s surroundings or from a position located away from the work area. However, solutions showing partial or full autonomy at work are being increasingly reported [4,5,6,7,8]. As the degree of autonomy for UGVs increases, human involvement in their control is being reduced. The “follow me” mode (classified as partial autonomy [9,10,11]) ensures that the operator does not need to manually control the platform. In this mode, the platform follows the human guide using a built-in sensor or set of sensors. In contrast to the teleoperation mode, where the operator manually controls the platform using a handheld or fixed control station, in the “follow me” mode, the UGV’s trajectory is generated by the human’s movement in the platform’s operating area. The most important tasks of this mode are maintaining a set distance from the guide and keeping the platform’s heading directed towards that guide. The platform needs to maintain a set safe distance from the guide and follow him or her in a smooth motion.

As mentioned before, the main task of the guide is to create a path for the platform. The guide must plan his or her route while keeping the specific platform’s characteristics in mind (e.g., different driving systems, such as tracked, wheeled, etc.). The guide does not have to move smoothly (he or she may stop during movements in case of emergency) and the system should be able to copy the guide’s path. The implementation of these tasks is possible using the components of the “follow me” system, including the guide’s (surroundings) observational subsystem, the trajectory planning subsystem, and the control subsystem. The observation of the guide (surroundings) is the basic component, with which the location of the moving guide (relative or absolute) is determined. Knowing the guide’s location is essential and represents the basis for the entire “follow me” application. This is the premise for the presented study on the guide’s location system for subsequently determining the desired trajectory of the UGV. The trajectory planning subsystem creates a path from successive guide positions. A trajectory of motion is then generated to connect two or more locations. Ultimately, the trajectory planning system aims to generate input signals to the control system that ensure smooth execution of the planned trajectory.

Applications utilizing the “following a guide” approach to control the movement of a UGV can be classified using different criteria, such as functionality, the used technologies, and the degree of autonomy. These systems have been used most prominently in industry, agriculture, and the military. Solutions with a higher degree of autonomy are much more technically and functionally complex than systems with a lower degree of autonomy. In these solutions, the platform does not rely as heavily on the guide’s movement (the guide only provides a “suggestion” regarding the direction of the platform’s movement). The platform independently determines its trajectory based on the information from several sensors (using complex algorithms involving artificial intelligence methods). The guide’s responsibility in creating the desired trajectory thus decreases, because the system independently generates a collision-free trajectory, which sometimes deviates from the guide’s path. For this to work, however, the system must have sufficiently high computing power to process large amounts of sensor data, generate the trajectory of movement, and determine a control signal for the platform during movement [11].

To observe the guide (surroundings), the following sensors are most commonly used: vision systems, laser rangefinders, ultrasonic sensors, satellite navigation systems, and wireless radio technologies, such as WI-FI, Bluetooth, and Ultra-wideband (UWB) [12,13,14,15,16,17,18,19,20,21]. Both vision systems and laser rangefinders lead in terms of popularity because they additionally enable the observation of the platform’s surroundings (including the guide) [12,13,14,15,16,17,18,19,20]. However, in the context of working in an outdoor environment, vision systems are highly exposed to unfavorable lighting conditions (uneven lighting and low visibility at night), while for laser rangefinders, the main problem is the presence of transparent (e.g., glass) or dark surfaces and certain weather conditions, such as rain. In the case of ultrasonic sensors, the phenomenon of mirror reflections and sensitivity to changes in ambient temperature can disrupt the proper operation of the sensors [21]. Satellite navigation systems, e.g., GPS, allow the receiver to have an absolute location but are exposed to deliberate signal interference (electronic interference and jamming), multipath signals (the signal may reflect on obstacles, like rocks), and the presence of terrain cover (e.g., forest), preventing location determination [22]. In turn, Ultra-wideband technology is the most promising technology among the previously mentioned wireless technologies, including WI-FI, Bluetooth, etc. UWB enables centimeter-level location services, low power consumption, interference immunity (it does not interfere with most of the existing radio systems), and high multipath immunity, with both LOS (Line of Sight) and NLOS (Non-Line of Sight) operations (signal propagation through objects) [23]. UWB technology has some disadvantages too. Since the UWB system is a low-power transmission system, its range is limited and strongly depends on the environment. Additionally, the possibility of interference with systems that operate in ultra-wide spectrums can be considered as a weakness of the technology (it depends on the country). Mentioned interference can also occur from the existing devices. The proximity of UWB modules to the human body can also negatively impact ranging accuracy because the human body is mostly composed of water. Additionally, design and implementation of UWB antennas can be problematic and UWB signal processing is very complex [23,24,25].

A popular trend in the design of “follow me” systems is sensor fusion, which focuses on combining several data streams from different sensors within one system [16,17,18,19,20]. The combined use of several such systems significantly increases the reliability of the described systems. Thus, it is reasonable to use UWB technology when developing a guide’s location system (which is a part of the “follow me” functionality). The presented solution utilizes four UWB modules, but the system allows for two to four UWB modules to be used for localization [26,27]. These units are placed at the corners of the robot’s frame. When three or more modules are used, the preferred localization algorithm is trilateration, while for two modules, geometric methods are used as a simplified trilateration model.

This article focuses on presenting the field test results of a developed outdoor localization system based on UWB technology. The elements of the developed subsystem were spread between being deployed on an existing UGV (four UWB modules) and on a guide equipped with mobile versions of the localization system. In the proposed application, the UWB technology works in a reverse configuration. This means that the elements whose positions are being measured are located outside of the area limited by the measuring elements.

This article describes the complete process of UWB signal processing, including its acquisition, the methods used for determining the guide’s location, and filtering. We examine the possibility of using modified versions of these selected algorithms for determining the location of the guide in the created location system using trilateration, methods for nonlinear programming, and a geometric algorithm proposed in this study. The conducted research is aimed at indicating which of the analyzed algorithms is best suited for this purpose. The modifications that we propose are characterized by the variable (conditional) structure of the above-mentioned algorithms, which were adapted to the number of signals from UWB sensors available for processing. The innovation of our study involves the implementation of an algorithm structure that changes the selection of equations (the mathematical model) used for determining the location of the guide depending on the measurement combination of UWB sensors.

## 2. Materials and Methods

**Figure 1:**
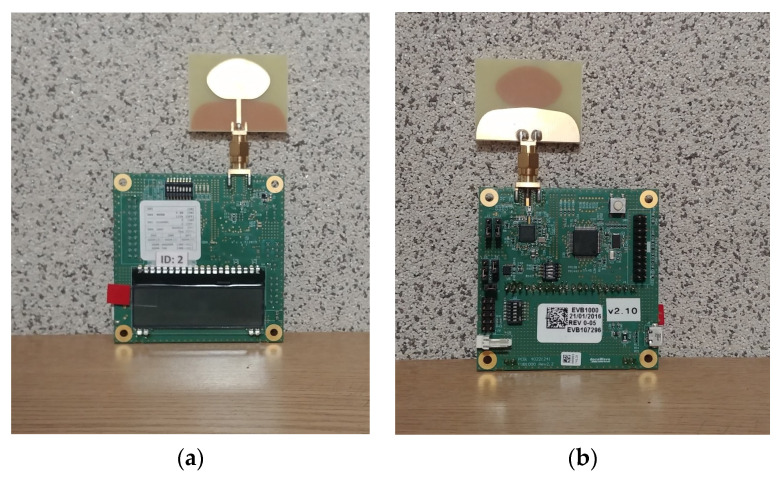
TREK1000 module: (**a**) front view and (**b**) rear view.

**Figure 2:**
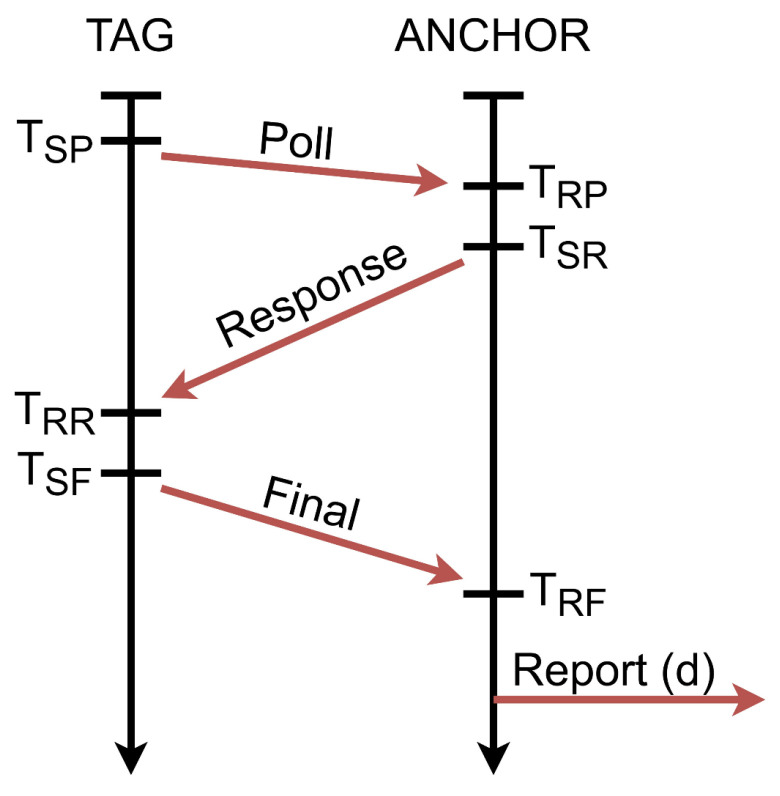
Diagram of communication between the tag and the anchor.

A UWB Decawave TREK 1000 (Figure 1) system was used in this research [28]. This system allows measuring the distance in a straight line between a portable transmitter called a tag and four receivers called anchors. The UWB modules use a two-way distance determination method called two-way ranging (TWR), whose operational diagram is shown in Figure 2 [29].

The parameters shown in Figure 2 (T_SP_, T_RP_, T_SR_, T_RR_, T_SF_, T_RF_) mean the following: the time of sending and receiving the polls and the packet responses by the anchor and the tag, respectively. Finally, the distance between the anchor to the tag can be determined using the following formula:

(1) $$d=c\left[\frac{\left(T_{\mathrm{RR}}-T_{\mathrm{SP}}\right)-\left(T_{\mathrm{SR}}-T_{\mathrm{RP}}\right)+\left(T_{\mathrm{RF}}-T_{\mathrm{SR}}\right)-\left(T_{\mathrm{SF}}-T_{\mathrm{RR}}\right)}{4}\right]$$

where c is the speed of light [29].

UWB modules are wireless distance sensors that return the distance in a straight line between a given anchor and a tag. This value is expressed in millimeters and updated with a frequency up to 10 Hz. The refresh rate is dependent on the configuration of the module’s operational band, which can be set between 3.5 and 6.5 GHz. The measurements are read via a USB port. At least one anchor is required to be connected to the computer to read the measurements.

**Figure 3:**
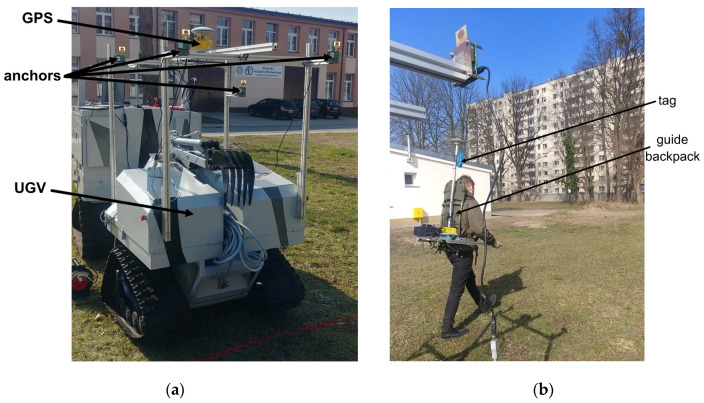
(**a**) Configuration of anchors on an unmanned ground vehicle (UGV), (**b**) moving guide.

The “Dromader” UGV (Figure 3a) was created at the Military University of Technology in Warsaw as part of the project ICAR of the European Defence Agency. Its main purpose is to support soldiers in crossing challenging terrain, in patrol and reconnaissance missions, as well as to search for dangerous materials in different terrain types.

The location system anchors were placed on the UGV in the configuration shown in Figure 3a. A special bracket was designed and manufactured, enabling easy and rapid reconfiguration of the localization system if needed. The tag was linked to the bracket attached to the guide’s backpack at an adjustable height above his head and turned towards his body (Figure 3b).

**Figure 4:**
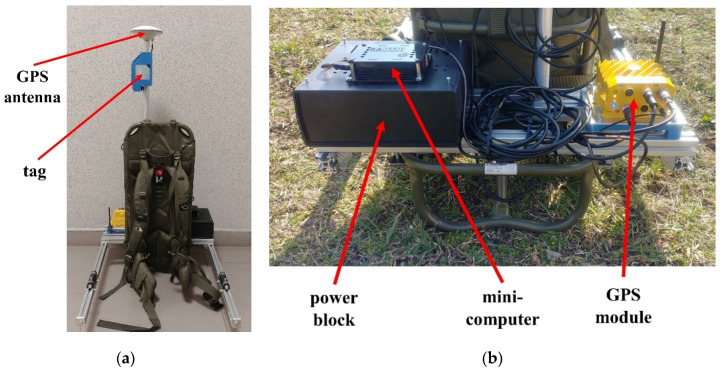
Guide backpack: (**a**) front view and (**b**) rear view.

To verify the location obtained using UWB technology, two SwiftNav DURO satellite receivers were used (hereafter abbreviated as GPS). The first one is used to determine the position of the guide (Figure 4a), while the second one determines the position of the selected UGV’s characteristic points (Figure 3a). The used receiver has access to GPRS packets through a built-in 3G modem. To receive real-time kinematic (RTK) corrections, the Leica Geosystems SmartNet network is accessed (GPS and/or GNSS receiver network via nationwide reference stations). Both receivers operate in RTK mode, which allows for high location accuracy (error: 1 cm horizontally and 1.5 cm vertically [30]).

For the UGV (Figure 3a), the GPS module with the antenna was placed on the UWB bracket, while for the guide, the elements were separated by placing the antenna on the UWB sensor’s axis (Figure 4a) and the GPS module on the back of the backpack (Figure 4b). These choices were dictated by facilitating the propagation of satellite signals to the receiver antennas. An additional application of these devices is the synchronization of measurements via the simultaneous recording of UTC time carried out for both UGV and the guide. The sampling frequency of the mentioned receivers was set to 10 Hz.

To ensure the required mobility of the guide, a power block was also placed on the backpack (Figure 4b). To record data from the GPS, a Raspberry Pi 3A minicomputer was used (Figure 4b). In the power block, there are two battery modules, one with a capacity of 20 Ah (the Raspberry Pi power supply, rated at 5 V DC) and the other with a capacity of 10 Ah (the GPS module power supply, rated at 12 V DC).

**Figure 5:**
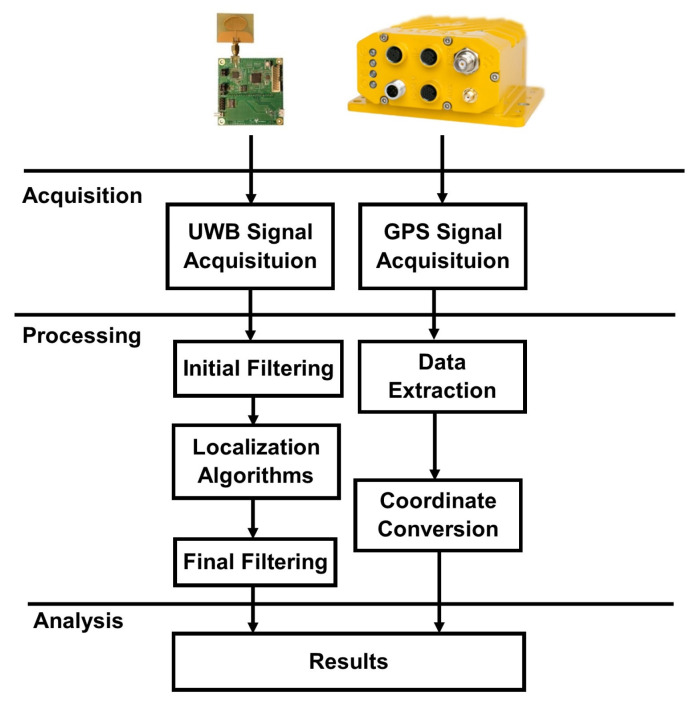
A detailed diagram of the data processing.

Data processing from the UWB modules and the GPS consists of several stages: acquisition, signal processing and analysis (Figure 5). The first mutual stage for these devices is data acquisition. UWB signal processing includes the initial filtering of distance measurements from the anchors, determination of the guide’s location using the localization algorithm, and the final filtering of the obtained location results (Figure 5). Each of these stages will be discussed in detail later in this article.

For the GPS, data acquisition is followed by data extraction (Figure 5). Two sets of data are recorded: the geodetic coordinates from the National Marine Electronics Association (NMEA) sentences (international standard for the formatting of Global Positioning System information) and UTC timestamps. To verify the guide’s path, the guide’s trajectory in a coordinate system expressed in units of the metric system is described. The geographical location derived from the NMEA sentences provides the geodetic coordinates: latitude, longitude, and altitude. Latitude and longitude are expressed in degrees, while altitude is expressed in meters.

**Figure 6:**
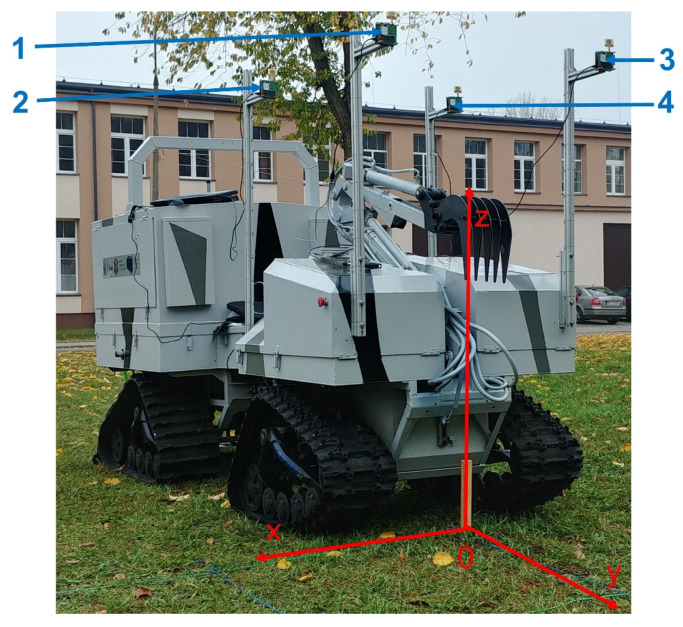
The unmanned ground vehicle with the anchors no. 1,2,3,4 and the adopted coordinate system.

One of the cartographic projection types using metric units is Universal Transverse Mercator (UTM), which is a universal rectangular flat coordinate system for the entire globe [31]. In addition, the vertical axis in the said system is consistent with the adopted axis of the UGV’s coordinate system shown in Figure 6. In the guide’s case, the GPS antenna and tag are collinearly arranged, which allows a subsequent comparison of the location of the guide using the GPS and localization algorithms.

Coordinate conversion of geodetic coordinates to the UTM system is the next stage of the GPS data processing (Figure 5). The last common stage for the two devices is an analysis of the results in a graphical data presentation (charts). All calculations were performed using the MATLAB/Simulink software.

### 2.1. Initial Filtering

A one-dimensional median filter with a window size of three was used for initial signal filtering. The median filter is a nonlinear filter that operates on the principle of selecting the middle value of an ascending sequence of values with the number of elements equal to the length of the window [32]. The premise behind the application of the filter is its ability to remove impulse interference from disturbed signals.

### 2.2. Localization Algorithms

Methods for determining the guide’s location are the basis for the operation of the developed localization system. In the analysis of the used localization algorithms, attention was paid to the Time of Arrival (TOA) method (the previously described TWR uses Time of Flight (TOF) of the UWB signals for distance calculation). For this purpose, among others, geometrical methods, trilateration, Taylor Series, extended Kalman filters, particle filters, or optimization methods can be used [33,34,35,36,37,38,39].

The minimum number of anchors necessary to locate the guide in the two-dimensional coordinate system is two (assuming that the guide is in front of the platform). This approach is the basis of the proposed geometric algorithm (GEO). Along with trilateration (TRI), these approaches constitute very clear and easy to implement localization methods. On the other hand, the method of nonlinear programming (NLP) allows the use of optimization methods to solve the problem of guide location and is much more computationally complex than the previous two methods.

Localization algorithms are adapted to the requirements of a specific solution (mostly accuracy) and have a specific number of inputs consistent with the hardware architecture of a given solution (in the case under consideration, four anchors). Data loss in wireless communication is a common phenomenon that limits the number of available measurements. If an algorithm is designed for a specific number of input signals, in the absence of one of the signals, the algorithm will not work properly, i.e., it will indicate a wrong location or the absence of a location. Therefore, we propose a modified version of the discussed algorithms (with a variable structure) that can be used as part of the location system. The basic mathematical dependencies are presented in Section 2.2.1, while in Section 2.2.2, Section 2.2.3, and Section 2.2.4, the basics of the aforementioned algorithms are presented.

#### 2.2.1. Basic Mathematical Dependencies

It is assumed that the tags and anchors are arranged in a three-dimensional xyz coordinate system at the following points (Figure 6):

- Tag: T (x_T_, y_T_, z_T_);
- Anchor no. 1: K_1_ (x_1_, y_1_, z_1_);
- Anchor no. 2: K_2_ (x_2_, y_2_, z_2_);
- Anchor no. 3: K_3_ (x_3_, y_3_, z_3_);
- Anchor no. 4: K_4_ (x_4_, y_4_, z_4_).

Knowing the individual distances between the tag and the ith anchor (i = 1, 2, 3, 4), the following formula can be written:

(2) $$d_{i}{}^{2}={\left(x_{T}-x_{i}\right)}^{2}+{\left(y_{T}-y_{i}\right)}^{2}+{\left(z_{T}-z_{i}\right)}^{2}$$

where d_i_ is the distance between the ith anchor and the tag (i = 1, 2, 3, 4).

This article considers the location of the guide in a two-dimensional xy coordinate system, which allows one to simplify Formula (2) into the following form:

(3) $$d_{1}{}^{2}={\left(x_{T}-x_{1}\right)}^{2}+{\left(y_{T}-y_{1}\right)}^{2}$$

(4) $$d_{2}{}^{2}={\left(x_{T}-x_{2}\right)}^{2}+{\left(y_{T}-y_{2}\right)}^{2}$$

(5) $$d_{3}{}^{2}={\left(x_{T}-x_{3}\right)}^{2}+{\left(y_{T}-y_{3}\right)}^{2}$$

(6) $$d_{4}{}^{2}={\left(x_{T}-x_{4}\right)}^{2}+{\left(y_{T}-y_{4}\right)}^{2}$$

where dependencies (3)–(6) describe the elementary geometrical relationships between the position of the UWB tag (point T) and the positions of the UWB anchors (points K_1_, K_2_, K_3_, K_4_) in a two-dimensional coordinate system.

#### 2.2.2. Trilateration

Dependencies (3)–(6) are nonlinear due to variables x_T_ and y_T_. The most common practice for linearizing nonlinear systems is to use the Taylor Series expansion of functions. In the case of dependencies (3)–(6), simple analytical transformations can lead to a linear system of equations in a closed-form. For this purpose, a reference anchor needs to be adopted (anchor no. 1). The next step is the pairwise subtraction of Equations (3)–(6) in the following way:

(7) $$d_{1}{}^{2}-d_{2}{}^{2}=2\left(x_{2}-x_{1}\right)x_{T}+2\left(y_{2}-y_{1}\right)y_{T}+x_{1}{}^{2}+y_{1}{}^{2}-x_{2}{}^{2}-y_{2}{}^{2}$$

(8) $$d_{1}{}^{2}-d_{3}{}^{2}=2\left(x_{3}-x_{1}\right)x_{T}+2\left(y_{3}-y_{1}\right)y_{T}+x_{1}{}^{2}+y_{1}{}^{2}-x_{3}{}^{2}-y_{3}{}^{2}$$

(9) $$d_{1}{}^{2}-d_{4}{}^{2}=2\left(x_{4}-x_{1}\right)x_{T}+2\left(y_{4}-y_{1}\right)y_{T}+x_{1}{}^{2}+y_{1}{}^{2}-x_{4}{}^{2}-y_{4}{}^{2}$$

where d_j_ is the distance between the jth anchor and the tag (j = 1, 2, 3, 4) [39].

If measurements from anchor no. 1 are not available, then Equations (7)–(9) are not met. Therefore, new analogous equations should be determined, this time using a different reference anchor (e.g., anchor no. 2):

(10) $$d_{2}{}^{2}-d_{3}{}^{2}=2\left(x_{3}-x_{2}\right)x_{T}+2\left(y_{3}-y_{2}\right)y_{T}+x_{2}{}^{2}+y_{2}{}^{2}-x_{3}{}^{2}-y_{3}{}^{2}$$

(11) $$d_{2}{}^{2}-d_{4}{}^{2}=2\left(x_{4}-x_{2}\right)x_{T}+2\left(y_{4}-y_{2}\right)y_{T}+x_{2}{}^{2}+y_{2}{}^{2}-x_{4}{}^{2}-y_{4}{}^{2}$$

If all (four) signals are available from the anchors, the following overdetermined system of linear Equations (7)–(9) with two unknowns x_T_ and y_T_ can be obtained:

(12) $$Y_{M}=A_{M}X_{M}$$

where

(13) $$Y_{M}=\left[\begin{array}{c} d_{1}{}^{2}-d_{2}{}^{2}+x_{1}{}^{2}+y_{1}{}^{2}-x_{2}{}^{2}-y_{2}{}^{2}\\ d_{1}{}^{2}-d_{3}{}^{2}+x_{1}{}^{2}+y_{1}{}^{2}-x_{3}{}^{2}-y_{3}{}^{2}\\ d_{1}{}^{2}-d_{4}{}^{2}+x_{1}{}^{2}+y_{1}{}^{2}-x_{4}{}^{2}-y_{4}{}^{2} \end{array}\right]$$

(14) $$A_{M}=\left[\begin{array}{cc} 2\left(x_{2}-x_{1}\right) & 2\left(y_{2}-y_{1}\right)\\ 2\left(x_{3}-x_{1}\right) & 2\left(y_{3}-y_{1}\right)\\ 2\left(x_{4}-x_{1}\right) & 2\left(y_{4}-y_{1}\right) \end{array}\right]$$

(15) $$X_{M}=\left[\begin{array}{c} x_{T}\\ y_{T} \end{array}\right]$$

The location of the tag can be determined using the least squares method (matrix $A_{M}$ is not invertible):

(16) $$X_{M}={\left(A_{M}{}^{T}A_{M}\right)}^{-1}A_{M}{}^{T}Y_{M}$$

However, in the case of three available signals from the anchors (where one signal is unavailable), the following system of linear equations can be obtained:

(17) $$Y_{N}=A_{N}X_{M}$$

where

(18) $$Y_{N}=\left[\begin{array}{c} d_{i}{}^{2}-d_{j}{}^{2}+x_{i}{}^{2}+y_{i}{}^{2}-x_{j}{}^{2}-y_{j}{}^{2}\\ d_{i}{}^{2}-d_{k}{}^{2}+x_{i}{}^{2}+y_{i}{}^{2}-x_{k}{}^{2}-y_{k}{}^{2} \end{array}\right]$$

(19) $$A_{N}=\left[\begin{array}{cc} 2\left(x_{j}-x_{i}\right) & 2\left(y_{j}-y_{i}\right)\\ 2\left(x_{k}-x_{i}\right) & 2\left(y_{k}-y_{i}\right) \end{array}\right]$$

(20) (i,j,k)∈{(1,2,3),(1,2,4),(1,3,4),(2,3,4)}

To determine the location of the tag, the following matrix transformation should be performed (det(A_N_) ≠ 0):

(21) $$X_{M}=A_{N}{}^{-1}Y_{N}$$

In addition, the value of the A_N_ matrix determinant depends on the arrangement of the anchors in the two-dimensional xy coordinate system [33].

**Figure 7:**
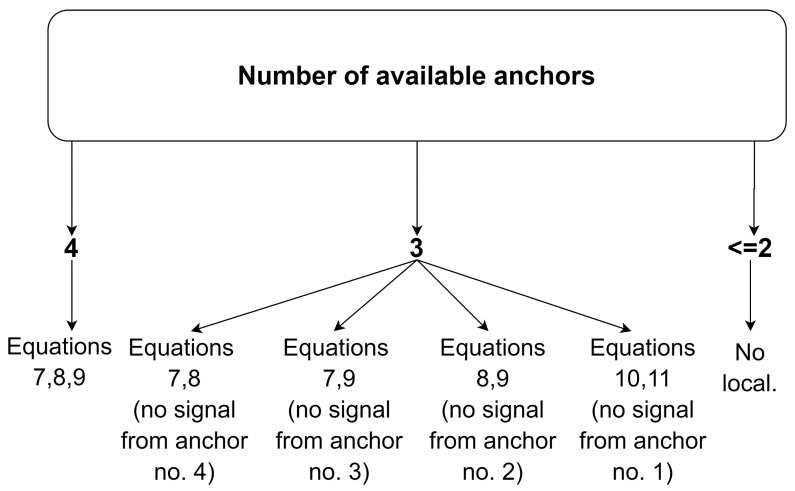
Conditional scheme of equation selection in the trilateration algorithm.

The proposed modified trilateration algorithm has a conditional structure depending on the number of measurements available from the anchors. The algorithm requires the availability of at least three anchors to determine the location, as shown schematically in Figure 7.

Under the availability of all measurements from the four anchors, the algorithm uses dependencies (7)–(9); however, in the case of no signal from one of the anchors, the algorithm uses dependencies from the other three anchors, e.g., when the signal from anchor 4 is not available, the algorithm determines the location from the dependencies (7)–(8) (Figure 7).

#### 2.2.3. Geometrical Method

**Figure 8:**
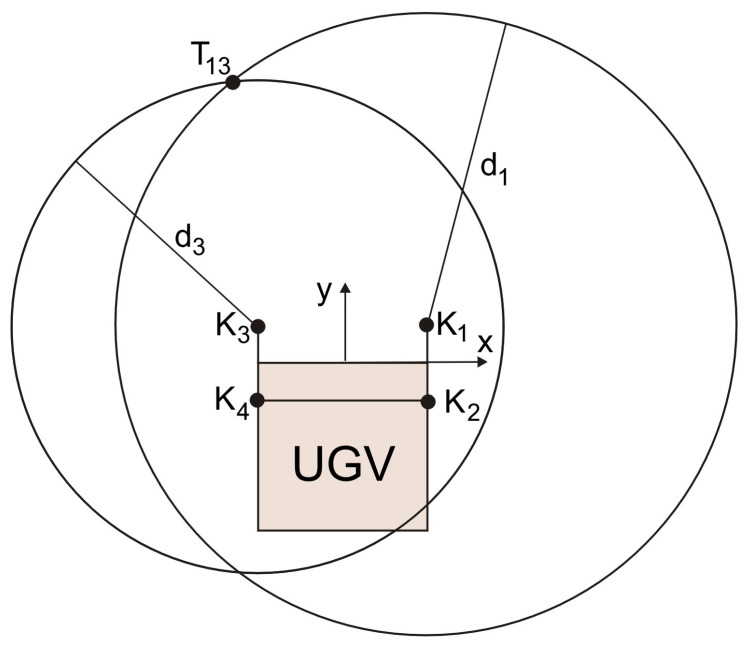
Determining the location of the guide with the use of anchors no. 1 and no. 3.

The geometric method is tailored to the proposed arrangement of the UWB modules on the UGV (Figure 6). It is based on TOA (Figure 8) and it is meant to provide a fast and computationally light way of determining the location of the guide [35].

The described method is based on dependencies (3)–(6), which are quadratic equations due to the two unknowns (x_T_ and y_T_). Moreover, it means that the location of the guide can be determined from two of them (two equations with two unknowns). A system of the two quadratic equations has two solutions, one of which should be neglected. After assuming that the guide is always in front of the UGV, the second solution (behind the UGV) can be neglected (Figure 8). Additionally, we cannot determine the location from any given pair of anchors due to their arrangement. It is possible only from four pairs of anchors ((1,3), (1,4), (2,3), and (2,4)), which are placed on the opposite sides of the UGV (Figure 8). In turn, pairs of anchors (1,2) and (3,4) are placed too close to each other, which causes numerical problems and leads to large localization errors [10,35].

Figure 8 shows the method of determining the location of a guide with the use of anchors no. 1 and no. 3 (point T_13_). In the case of other pairs of anchors, the method of determining the location of the guide is analogous.

Under no loss of measurements from the modules, four locations (from four pairs of anchors: (1,3), (1,4), (2,3) and (2,4)) can be obtained, where the weighted arithmetic mean is the final location of the guide [x_T_, y_T_]^T^:

(22) $$x_{T}=\frac{w_{1}x_{T13}+w_{2}x_{T14}+w_{3}x_{T23}+w_{4}x_{T24}}{w_{1}+w_{2}+w_{3}+w_{4}}$$

(23) $$y_{T}=\frac{w_{1}y_{T13}+w_{2}y_{T14}+w_{3}y_{T23}+w_{4}y_{T24}}{w_{1}+w_{2}+w_{3}+w_{4}}$$

where [x_Tij_, y_Tij_]^T^ is the location of the guide determined from pair of (i, j) anchors and w_k_ is non-negative weights (∑w_k_ = 1), k = 1,…, 4.

Under the loss of signal from, e.g., anchor no. 1, the final location of the guide can be determined from two locations (two pairs of anchors (2,3) and (2,4)):

(24) $$x_{T}=\frac{w_{11}x_{T23}+w_{12}x_{T24}}{w_{11}+w_{12}}$$

(25) $$y_{T}=\frac{w_{11}y_{T23}+w_{12}y_{T24}}{w_{11}+w_{12}}$$

where w_1k_ is non-negative weights (∑w_1k_ = 1), k = 1,…, 2.

Finally, under the loss of signals from, e.g., anchors no. 1 and no. 3, the final location of the guide can be determined from only one location (one pair of anchors (2,4)):

(26) $$x_{T}=x_{T24}$$

(27) $$y_{T}=y_{T24}$$

In case of loss of signals from other anchors, the dependencies ((24)–(25) or (26)–(27)) describing the location of the guide are analogous.

**Figure 9:**
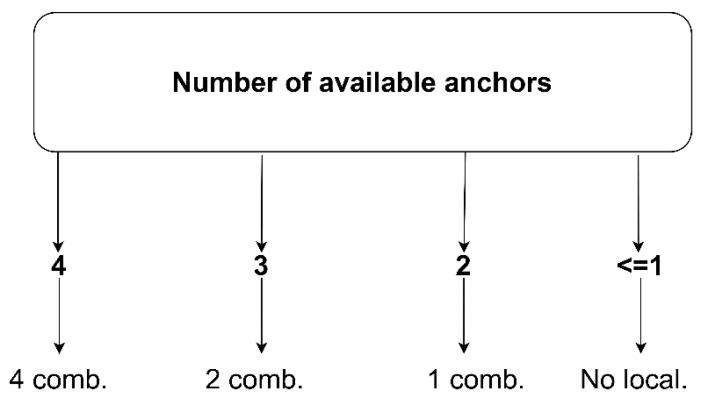
The number of combinations of anchor pairs used in localization under the geometrical method.

Figure 9 shows a diagram describing the number of combinations of anchor pairs from which the guide location is determined in the case of limited measurement availability from the UWB sensors. Additionally, weights were obtained using numerical simulations.

Localization with the use of the geometrical method is impossible if at least three out of four signals from the UWB modules are lost (Figure 9).

#### 2.2.4. Nonlinear Programming

Nonlinear programming (NLP) is the process of solving an optimization problem in which some constraints or objective functions are nonlinear [36]. The considered problem of determining the location of the guide can be presented as follows. First, a set of four nonlinear relationships F_i_(x) (i = 1, 2, 3, 4) is given:

(28) $$F_{1}\left(x\right)=\sqrt{{\left(x_{T}-x_{1}\right)}^{2}+{\left(y_{T}-y_{1}\right)}^{2}}-d_{1}.$$

(29) $$F_{2}\left(x\right)=\sqrt{{\left(x_{T}-x_{2}\right)}^{2}+{\left(y_{T}-y_{2}\right)}^{2}}-d_{2}.$$

(30) $$F_{3}\left(x\right)=\sqrt{{\left(x_{T}-x_{3}\right)}^{2}+{\left(y_{T}-y_{3}\right)}^{2}}-d_{3}.$$

(31) $$F_{4}\left(x\right)=\sqrt{{\left(x_{T}-x_{4}\right)}^{2}+{\left(y_{T}-y_{4}\right)}^{2}}-d_{4}.$$

The solution for the given set of equations is the vector x = [x_T_, y_T_]^T^, which solves the equations F_i_(x) = 0 (i = 1, 2, 3, 4). For this purpose, it uses the Levenberg–Marquardt algorithm (LM), which is one of the most commonly used iterative, gradient, and nonlinear optimization algorithms [40].

This algorithm seeks to solve the set of above equations by minimizing the following cost function f(x):

(32) $$\underset{x}{\min}f\left(x\right)=\overset{4}{\underset{i}{\sum }}F_{i}{}^{2}\left(x\right)$$

The LM algorithm uses a search direction which is a solution of the linear set of equations:

(33) $$\left(J{\left(x_{k}\right)}^{T}J\left(x_{k}\right)+\mu_{k}I\right)d_{k}=-J{\left(x_{k}\right)}^{T}F\left(x_{k}\right)$$

where J(x_k_) is the Jacobian matrix of F(x_k_), at each iteration k, d_k_ is the vector of search direction and μ_k_ is the scalar damping parameter of the search [41].

**Figure 10:**
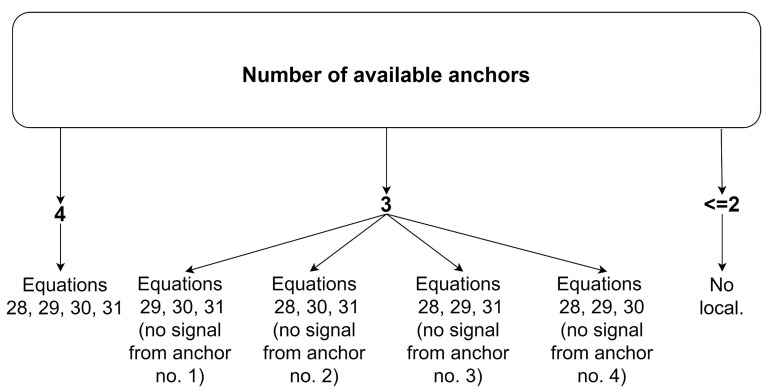
Conditional scheme of equation selection for cost function f(x) in nonlinear programming algorithm.

A characteristic feature of the described algorithm is its fast convergence of the solution resulting from a combination of the gradient descent and Gauss–Newton methods. The algorithm requires the initial vector x_0_ = [x_0_, y_0_]^T^, from which the search for a solution begins [39,40,41]. The algorithm requires the availability of at least three anchors (as in the case of the trilateration algorithm) to determine the location, as shown schematically in Figure 10.

In turn, under the loss of signal from one of the anchors, cost function f(x) is the sum of the squares of the three equations presented in Figure 10.

Under the loss of signal from, e.g., anchor no. 1, relationship (28) is not valid, so the analyzed algorithm seeks to solve the set of three remaining valid Equations (29)–(31) by minimizing the function f(x) in the following form:

(34) $$\underset{x}{\min}f\left(x\right)=F_{2}{}^{2}\left(x\right)+F_{3}{}^{2}\left(x\right)+F_{4}{}^{2}\left(x\right)$$

In case of loss of signal from another anchor, dependencies describing the function f(x) are analogous (Figure 10).

### 2.3. Final Filtering

A moving average filter with a window size of three was used for filtering the tag position series in time x_T_(t), y_T_(t). This is a linear filter used to filter signals in which information is coded in the time domain [30].

## 3. Results

The human guide travels along seven rectilinear trajectories inclined at different angles to the x axis of the adopted coordinate system. After reaching the turning point, the guide rotates 180° around their axis and makes a return movement along the same trajectory to his starting point.

The following coefficients were used to evaluate the obtained results of the tested algorithms:

- Total locational error:
  (35) $$e_{c}\left(t\right)=\sqrt{e_{x}{\left(t\right)}^{2}+e_{y}{\left(t\right)}^{2}}$$
  where e_x_(t) is the location tracking error on the x axis of the coordinate system at time t, and e_y_(t) is the location tracking error on the y axis of the coordinate system at time t.
- Quality indicator:
  (36) $$Q=\sum \left|e_{c}\left(t\right)\right|$$

**Figure 11:**
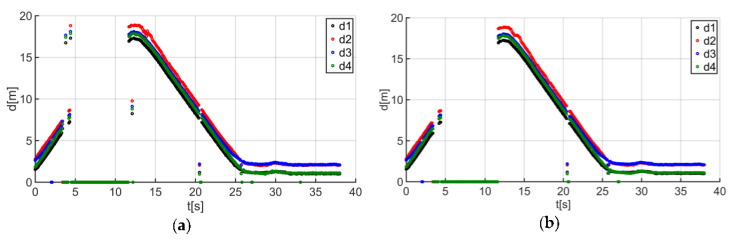
Comparison of the distance measurement results from anchors d_i_(t), (i = 1, 2, 3, 4): (**a**) before filtering and (**b**) after filtering for trajectory no. 3.

The first research stage was data acquisition for the location system. For this purpose, the guide covered the test track described in the paper. The obtained results showed that the created UWB system is susceptible to random signal decays, which was indicated by the sensor’s distance indications for one of the considered trajectories (Figure 11a).

The next step was to apply the initial filtering using a median filter with a window size of three. This enabled the removal of some outliers and improved signal continuity (Figure 11b). However, due to signal loss over 4–12 s, the median filter was used only to prepare it for further processing (signal pre-processing). The filter window size was determined based on numerical tests.

The next step was the introduction of data processing mechanisms in the form of TRI, NLP, and GEO. These mechanisms allowed us to determine the guide’s location in the described coordinate system.

**Figure 12:**
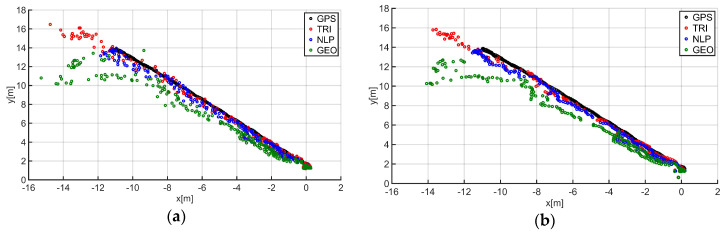
Comparison of the guide location results: (**a**) before final filtering and (**b**) after final filtering.

The final step was to perform the final filtering of the data by applying a moving average filter with a window size of three to the previously obtained location results. As in the case of the initial filtering, the filter window size was determined based on numerical tests. The location test results before and after filtering are shown in Figure 12a,b.

**Figure 13:**
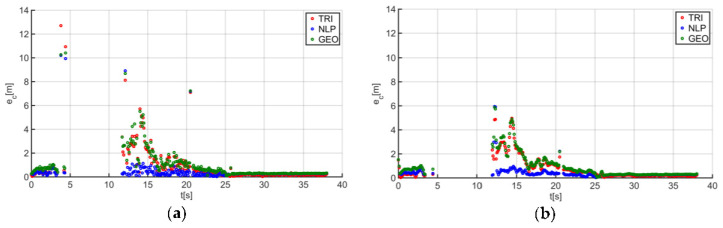
Comparison of the results of the total e_c_(t) location errors: (**a**) before final filtering and (**b**) after final filtering for trajectory no. 3.

The combination of the median and moving average filters in the case of the analyzed trajectory allowed us to reduce the total errors (Figure 13a,b) and improved the values of the quality indicator by 9.38% in the case of TRI, by 7.75% for NLP, and by 7.14% for GEO.

**Figure 14:**
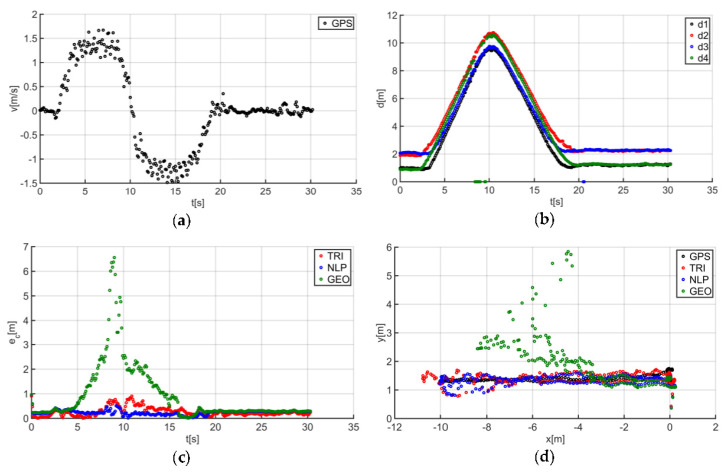
Test results no. 1: (**a**) human guide speed v_p_(t) and (**b**) filtered distance from anchors d_i_(t), i = 1, 2, 3, 4; (**c**) course of total location errors e_c_(t) and (**d**) location of the human guide y_p_(t) = f(x_p_(t)).

**Figure 15:**
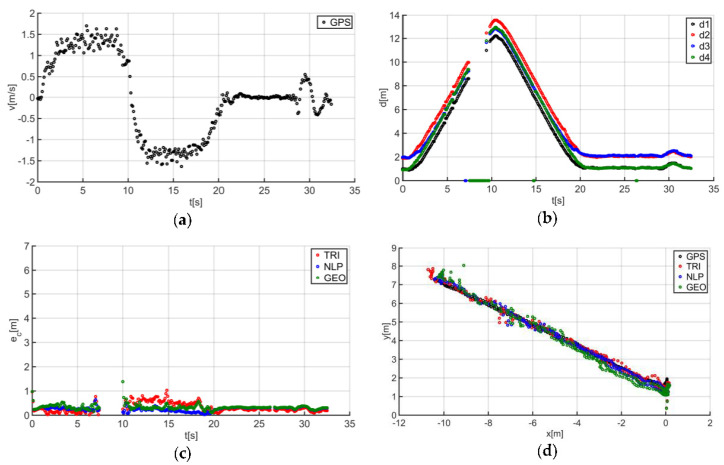
Test results no. 2: (**a**) human guide speed v_p_(t) and (**b**) filtered distance from anchors d_i_(t), i = 1, 2, 3, 4; (**c**) course of total location errors e_c_(t) and (**d**) location of the human guide y_p_(t) = f(x_p_(t)).

**Figure 16:**
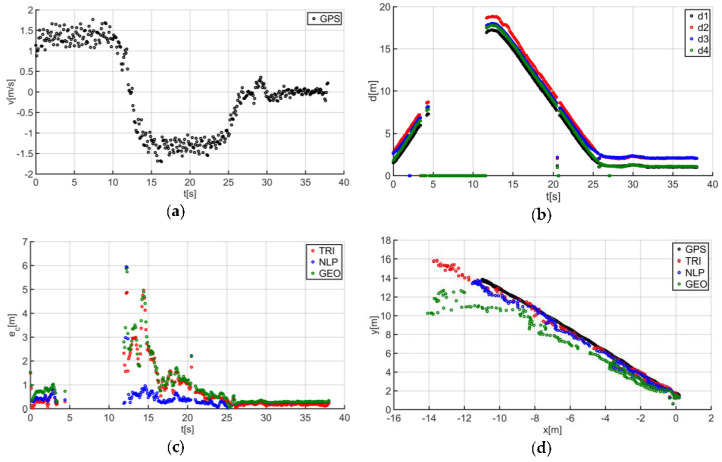
Test results no. 3: (**a**) human guide speed v_p_(t) and (**b**) filtered distance from anchors d_i_(t), i = 1, 2, 3, 4; (**c**) course of total location errors e_c_(t) and (**d**) location of the human guide y_p_(t) = f(x_p_(t)).

**Figure 17:**
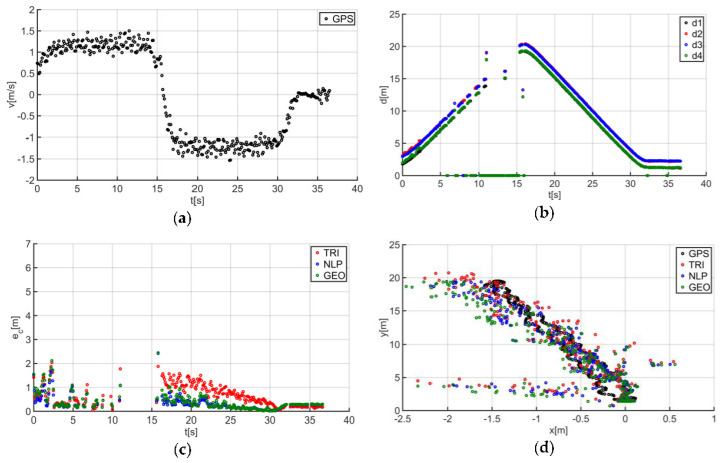
Test results no. 4: (**a**) human guide speed v_p_(t) and (**b**) filtered distance from anchors d_i_(t), i = 1, 2, 3, 4; (**c**) course of total location errors e_c_(t) and (**d**) location of the human guide y_p_(t) = f(x_p_(t)).

**Figure 18:**
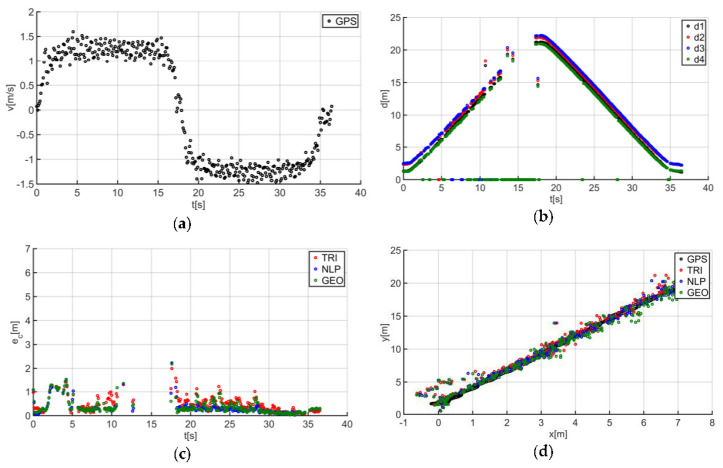
Test results no. 5: (**a**) human guide speed v_p_(t) and (**b**) filtered distance from anchors d_i_(t), i = 1, 2, 3, 4; (**c**) course of total location errors e_c_(t) and (**d**) location of the human guide y_p_(t) = f(x_p_(t)).

**Figure 19:**
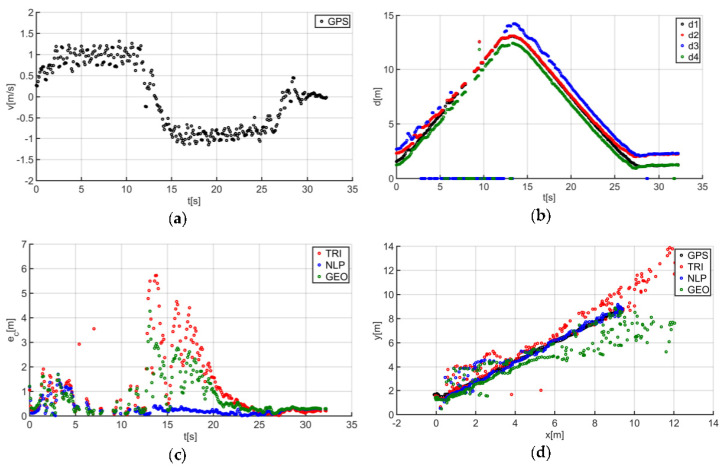
Test results no. 6: (**a**) human guide speed v_p_(t) and (**b**) filtered distance from anchors d_i_(t), i = 1, 2, 3, 4; (**c**) course of total location errors e_c_(t) and (**d**) location of the human guide y_p_(t) = f(x_p_(t)).

**Figure 20:**
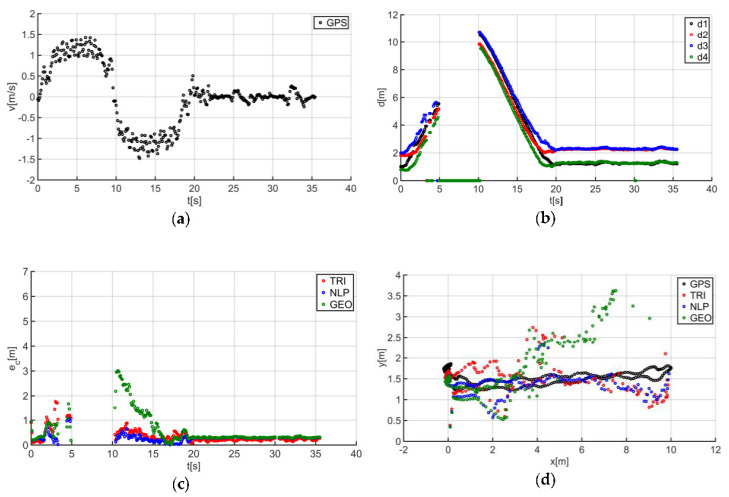
Test results no. 7: (**a**) human guide speed v_p_(t) and (**b**) filtered distance from anchors d_i_(t), i = 1, 2, 3, 4; (**c**) course of total location errors e_c_(t) and (**d**) location of the human guide y_p_(t) = f(x_p_(t)).

The described solutions were used for all the analyzed trajectories, which allowed us to obtain the results to be used in the last stage of data analysis. The use of a person as a guide during the tests enabled us to record results that are more similar to those found in actual “follow me” applications. Figure 14a presents the human guide’s speed signal determined using the GPS. This signal was used during processing to determine when the guide reached the key points of the trajectory (namely, the starting and the ending positions on the trajectory). For trajectory no. 1, the guide’s movement started at the 2nd second of the recorded signal and ended at the 20th second (Figure 14a). The results obtained as a result of using initial filtering methods are shown in Figure 14b. Signal loss locations are observable on the waveform. For these events, the distance between the tags and anchors dropped to 0. It should be noted that this only occurred when the guide moved away from the UGV. Signal loss had a negative impact on the accuracy of the location determination using the TRI, NLP, and GEO methods (Figure 14c), whereas the TRI and NLP methods generated positioning errors at a level of about 1 m maximum. The GEO method generated the largest total positioning errors (maximum of 6.5 m). The results of the TRI, NLP, and GEO methods applied were plotted based on the GPS’s output position for trajectory no. 1 (Figure 14d), for comparative purposes. Similarly, the results for trajectories no. 2, 3, 4, 5, 6, and 7 are presented in Figure 15, Figure 16, Figure 17, Figure 18, Figure 19 and Figure 20.

**Figure 21:**
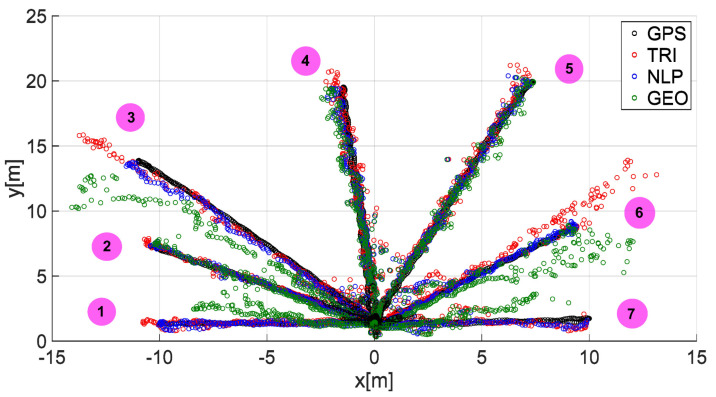
The results of the guide location for trajectories 1–7.

The speed of the human guide for all analyzed trajectories did not exceed 1.81 m/s (Figure 14a, Figure 15a, Figure 16a, Figure 17a, Figure 18a, Figure 19a and Figure 20a). In addition, for all the considered cases, repeated distance signal losses were observed in the first phase of the movement, i.e., moving away from UGV (Figure 14b, Figure 15b, Figure 16b, Figure 17b, Figure 18b, Figure 19b and Figure 20b). During turning and the second phase of the movement, this phenomenon occurred sporadically. Single signal losses increased the total location errors (Figure 14c, Figure 15c, Figure 16c, Figure 17c, Figure 18c, Figure 19c and Figure 20c), while for the signals lost from all anchors, guide localization was impossible. The total error values were omitted in these cases. The obtained location results from the use of the TRI, NLP, and GEO methods are shown in Figure 14d, Figure 15d, Figure 16d, Figure 17d, Figure 18d, Figure 19d, and Figure 20d. Figure 21 shows the results of both methods and (for reference purposes) the signals recorded from the GPS module for all tested human guide trajectories.

**Figure 22:**
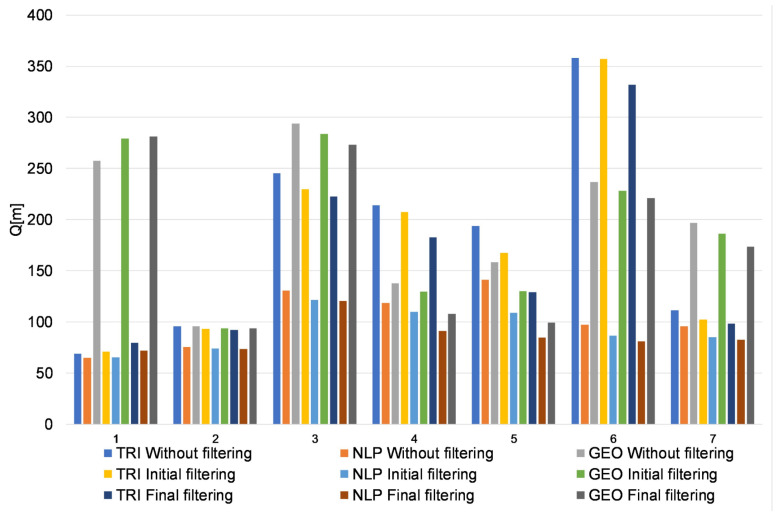
Results of the quality indicators Q for trajectories 1–7.

In addition, to qualitatively summarize the location results, Figure 22 presents the results of the quality indicators for TRI, NLP, and GEO for all the analyzed trajectories at each stage of signal processing.

The data presented in Figure 22 show that the use of initial filtering improved the location results in the case of TRI by 4.65%, for NLP by 9.93%, and for GEO by 3.33%. In turn, the use of final filtering (median filter and moving average filter) improved the location results in the case of TRI by 11.83%, for NLP by 16.33%, and for GEO by 9.22%.

**Figure 23:**
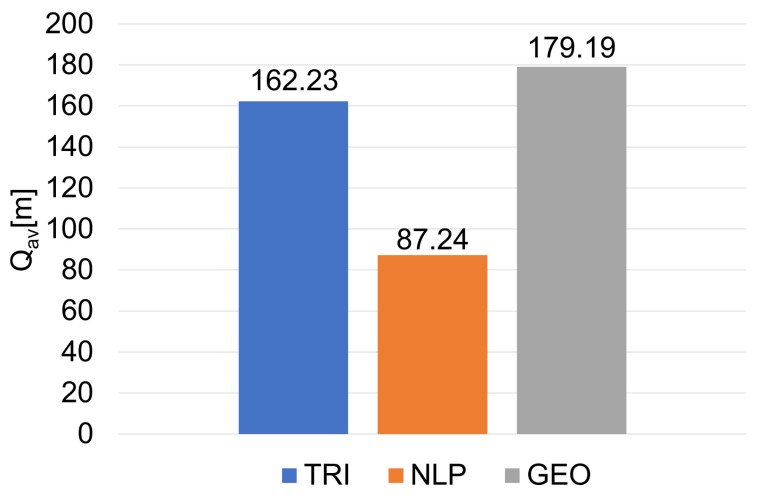
Results of the average quality indicators Q for localization algorithms (final filtering).

**Figure 24:**
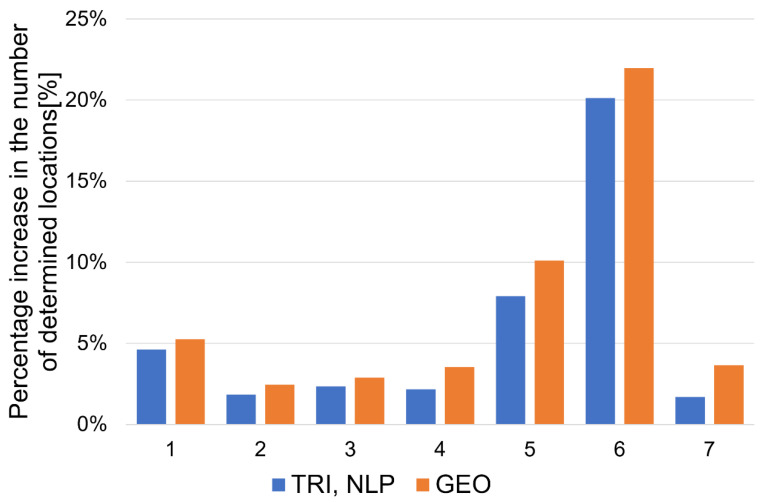
Percentage increase in the number of determined locations in the case of localization algorithms for trajectories 1–7.

Figure 23 shows the average values Q_av_ of the quality indicators Q for the final filtering, which were determined from all analyzed trajectories. Additionally, due to the applied modified versions of the localization algorithms, the percentage increase in the number of determined locations in relation to the total number of locations was also determined in cases where measurements were not available from all UWB modules (Figure 24).

Because the quality indicator Q is the sum of the absolute total location errors (36), and the desired localization algorithm should have minimal location errors, the average value of the quality indicator for the selected algorithm should also be minimal. The lowest value of the average quality indicator Q_av_ was obtained by the NLP (87.24 m). A value of about 1.9 times higher than the analogous indicator (162.23 m) was obtained by the TRI, and the value (179.19 m) obtained by the GEO was about 2.1 times higher than that obtained by the NLP.

The use of a modified algorithm structure allowed us to increase the percentage number of the determined locations (Figure 24) from about 2% (TRI, NLP—trajectory no. 2) to about 22% (GEO—trajectory no. 6).

## 4. Conclusions

In the case of the first phase of the guide’s movement (moving away from UGV), for each of the tested trajectories, periods of signal loss were observed (Figure 14a, Figure 15a, Figure 16a, Figure 17a, Figure 18a, Figure 19a and Figure 20a). However, this phenomenon was not observed on such a scale in the second phase of the movement (approaching UGV). The presumed reason for this phenomenon is obstruction of the transmitting antenna at the guide’s position by the mounting bracket of the UWB module. The research has shown that the tag should face the UGV to minimize signal losses.

The applied filtering (median filter and moving average filter) improved the location results for TRI by 11.83%, for NLP by 16.33%, and for GEO by 9.22%. However, the use of the aforementioned filtering in some cases (trajectory no. 1, Figure 22) may lead to a distortion of the original signal (the application of a median filter for a signal with a small number of impulse disturbances, Figure 14b) and, consequently, to an increase in localization errors.

The proposed modified structure of the location algorithms, adapted to the hardware structure of the solution (four UWB anchors), maximizes the number of determined locations depending on the number of available measurements from the anchors. The proposed TRI and NLP algorithms achieved an average percentage increase in the number of determined locations of about 5.8% (and of about 7.1% for GEO). The proposed system’s structure is universal and can be extended to other positioning algorithms since the basic geometric relationships between the tag and anchor positions do not change.

The NLP algorithm obtained the lowest value of the average quality index among all the analyzed algorithms, which means that NLP is the most accurate localization method considered in this article. The remaining algorithms obtained much higher values for the index (TRI—1.9 times higher; GEO—2.1 times higher), which translates into greater errors in the localization of the guide.

The low computational complexity of the TRI and GEO algorithms means that they can be used as generators of the initial location vector, which can be used as the starting point for detection via the NLP algorithm. This approach reduces the number of iterations needed to find the final solution.

The occurrence of noise in the distance measurements has a negative and observable impact on the localization accuracy of all considered algorithms (Figure 21). However, the noise has the greatest impact on the location accuracy of the GEO algorithm (Figure 22) compared to the other considered algorithms (it peaks in trajectories no. 1, 3, and 6), which results from the mathematical model of the algorithm (i.e., determining the location only from two measurements). For the NLP and TRI algorithms, the position is determined from a minimum of three measurements.

One possible direction of future research is to study the UGV’s planning trajectory subsystem.
